# Development of a preoperative prognostic scoring system to predict benefits of hepatic resection in advanced hepatocellular carcinoma patients

**DOI:** 10.1042/BSR20201928

**Published:** 2021-04-09

**Authors:** Chang-Zhi Chen, Jian-Hong Zhong, Ya-Peng Qi, Jie Zhang, Tao Huang, Liang Ma, Le-Qun Li, Tao Peng, Bang-De Xiang

**Affiliations:** 1 Hepatobiliary Surgery Department, Guangxi Liver Cancer Diagnosis and Treatment Engineering and Technology Research Center, Guangxi Medical University Cancer Hospital, Nanning, China; 2 Hepatobiliary Surgery Department, Guangxi Zhuang Automonous Region People Hospital, Nanning, China; 3 Hepatobiliary Surgery Department, The First Affiliated Hospital of Guangxi Medical University, Nanning, China

**Keywords:** Advanced hepatocellular carcinoma, Hepatic resection, Preoperative, Prognostic scoring system

## Abstract

Objective: The present study aimed to identify risk factors for overall survival in advanced hepatocellular carcinoma (HCC) patients and establish a scoring system to select patients who would benefit from hepatic resection.

Methods: Survival curves were analyzed using the Kaplan–Meier method and log-rank test. The prognostic scoring system was developed from training cohort using a Cox-regression model and validated in a external validation cohort

Results: There were 401 patients in the training cohort, 163 patients in the external validation cohorts. The training cohort median survival in all patients was 12 ± 1.07 months, rate of overall survival was 49.6% at 1 year, 25.0% at 3 years, and 18.0% at 5 years. A prognostic scoring system was established based on age, body mass index, alkaline phosphatase, tumor number and tumor capsule. Patients were classified as low- risk group(≤3.5) or high-risk group(>3.5). High-risk patients had a median survival of 9 months, compared with 23 months in low-risk patients. The area under the receiver operating characteristic curve (AUC) of the prognostic scoring system was 0.747 (0.694–0.801), which is significantly better than AFP, Child-Pugh and ALBI. The AUC of validation cohorts was 0.716 (0.63–0.803).

Conclusion**:** A prognostic scoring system for hepatic resection in advanced HCC patients has been developed based entirely on preoperative variables. Patients classified as low risk using this system may experience better prognosis after hepatic resection.

## Introduction

Hepatocellular carcinoma (HCC) is a common malignant liver cancer, and at the time of diagnosis, many patients are already in an advanced stage [1,2]. Advanced HCC, also known as Barcelona clinic liver cancer stage C disease, involves macrovascular invasion, or distant metastases or their combinations [3]. Both the European Association for the Study of the Liver (EASL) and the American Association for the Study of Liver Diseases (AASLD) recommend systemic therapy for patients with advanced HCC, which may extend survival by approximately 10 months [4,5]. However, progression of intrahepatic tumors is the cause of death in the majority of advanced HCC patients [6]. Therefore, active control or management of intrahepatic lesions may be an effective method to improve patient survival. A large systematic review showed that patients with advanced HCC who underwent hepatic resection had a longer survival time than those who underwent Chemoembolization [7], with a median survival time of 6–54 months [7–9]. Our previous study also demonstrated that hepatic resection has certain advantages over tace for patients with advanced HCC [10]. Combined with systemic treatment or TACE had a better outcome after resection [11].

In this way, hepatic resection has become a routine treatment for many advanced HCC patients in many geographic areas [9,12]. However, whether hepatic resection, systemic therapy or TACE can provide longer survival for specific subgroups of advanced HCC patients remains unclear [13–15]. Given that advanced HCC patients face high incidence of postoperative recurrence and metastasis, survival following resection may depend to a large extent on risk factors of recurrence, which include macrovascular invasion, lymph node metastasis, multiple tumors, incomplete capsule and higher alpha-fetoprotein (AFP) [16,17].

The present study explored the possibility of developing a prognostic model to predict which advanced HCC patients are more likely to experience overall survival benefit after resection. These efforts were inspired by other published prognostic models for patients with advanced HCC. One model has been developed to predict benefits of surgery to treat HCC involving portal vein tumor thrombus (PVTT) [18]; PVTT is a poor prognostic factor for HCC patients, and the median survival time ranges from 2.7 to 4.0 months without treatment. For resectable tumors, the treatment of HCC with PVTT should be hepatectomy and removal of PVTT. For those unresectable tumors, TACE has been the preferred treatment [19], this model is based on levels of albumin, AFP, and tumor diameter and number. Another model has been developed to predict the benefits of unresectable HCC. A six-and-twelve model was constructed based on the diameter and number of tumors, the patients were divided into three groups that according to the sum of tumor size and number ≤6, >6 but ≤12, and >12, and instructed in subsequent TACE treatment [20]. The present study aimed to establish a postoperative model to predict prognosis in advanced HCC patients based on preoperative variables. This model may help guide treatment for advanced HCC.

## Methods

### Patients and study design

The pathological and clinical data of patients with advanced HCC who underwent hepatectomy in the Guangxi Medical University Cancer Hospital were retrospectively analyzed from January 2006 to December 2016 and set as the training cohort. The validation cohort collected partial preoperative data and follow-up information of patients with advanced hepatocellular carcinoma who underwent hepatectomy in the First Affiliated Hospital of Guangxi Medical University from 2009 to 2020. The study was approved by the Institutional Ethics Committee of the two hospitals. Written informed consent was obtained from all the patients for their data to be used for research.

#### Purposes

Patients were included if they (1) were undergoing initial hepatectomy; (2) were diagnosed with HCC by postoperative pathology; (3) were diagnosed with advanced stage HCC involving macrovascular invasion or extrahepatic spread; and (4) had no preoperative antitumor therapy, including transarterial chemoembolization, local ablation, chemotherapy, radiotherapy or targeted therapy. Patients were excluded if they had extensive intraperitoneal implantation and dissemination, superior mesenteric vein tumor thrombus, other malignant tumor in the previous 5 years, or incomplete data.

### Standard definition

Continuous data for the following variables were converted to categories based on clinical criteria: AFP, aspartate aminotransferase (AST), alanine transaminase (ALT), total bilirubin (TBIL), albumin (ALB), prothrombin time (PT) and BMI. Continous data for the following variables were converted to categories based on the Youden index: age, white blood cell (WBC), red blood cell (RBC), hemoglobin (HB), platelet (PLT), alkaline phosphatase (ALP), gamma-glutamyl transpeptidase (GGT), and albumin-bilirubin (ALBI).

### Surgical treatment for advanced HCC

All patients underwent open surgery, complete resection of intrahepatic lesions was performed under visual observation in all patients. PVTT and hepatic venous tumor thrombus (HVTT) were resected. Patients with lymph node or intraperitoneal metastasis underwent regional or local resection. All intrahepatic lesions were completely resected in 20 patients with pulmonary metastasis. Extraperitoneal metastases were not treated during surgery.

### Follow-up

Patients were followed up by telephone or outpatient service. Patients were re-examined every 3 months within 2 years after hepatic resection and every 6 months after 2 years. Liver function, AFP, abdominal ultrasound and enhanced CT or MRI of the upper abdomen were performed.

### Study endpoints

OS was the primary endpoint in the present study, which was defined as interval from the first surgery to the data of death or to the end of follow-up. Patients who were alive at the end of follow-up were censored on January 31, 2019.

### Statistical analysis

The primary outcome was overall survival. SPSS 22.0 (IBM, Chicago, IL, U.S.A.) was used for statistical analysis. All graphs were generated using GraphPad Prism version 8.0.2 (GraphPad-Prism Software Inc., San Diego,CA, U.S.A.). Cox regression model was used for univariate and multivariate analysis. Survival rate was analyzed by the Kaplan–Meier method and log-rank test. Differences were considered significant when *P*<0.05. The multivariate Cox regression coefficient (β) was used to establish a prognostic point model. We has referred to Transparent Reporting of a multivariable prediction model for Individual Prognosis or Diagnosis (TRIPOD): the TRIPOD statement in our study [21].

## Results

### Patient characteristics

Initially, 422 cases were included. Patient data were excluded in 21 cases due incomplete data at follow-up. A total of 401 patients with advanced HCC were included in the training cohort, comprising 358 males and 43 females, with a median age of 47 years (range: 18–77 years). In the extrahepatic metastasis group, there were 20 cases of lung metastasis, 28 cases of lymph node metastasis and 1 case of adrenal metastasis (Table 1). The validation cohort included 162 patients with age, BMI, ALP, tumor capsule, tumor number, survival status and survival time (Table 2).

**Table 1:** Clinico-demographic characteristics and univariate and multivariate analyses of 401 patients with advanced hepatocellular carcinoma

Variables	*N*=401	*P* in univariate analysis	Multivariate analysis	
			*P*	HR (95%CI)
Gender (male/female)	358/43 (89/11%)	0.527		
Age (≥50/<50 years)	47 (18–77)	0.013	0.047	1.274 (1.003–1.617)
Body mass index (≥18.5/<18.5)	21.6 (14.9–33.6)	0.031	0.007	1.595 (1.138–2.236)
Diabetes (yes/no)	40/361 (10/90%)	0.753		
PVTT (yes/no)	310/91 (77.3/22.7%)	0.497		
HVTT (yes/no)	58/343 (14.5/85.5%)	0.818		
Extrahepatic metastasis (yes/no)	49/352 (12.2/87.8%)	0.171		
HBsAg (positive/ negative)	353/48 (88/12%)	0.936		
PT (≥13/<13 s)	12.9 (10–20)	0.670		
TBIL (≥17.1/<17.1 μmol/l)	12.0 (2.4–66.8)	0.029		
Albumin (≥40/<40 g/l)	39.8 (23–70)	0.938		
ALT (≥40/<40 U/l)	39 (7–292)	0.009	0.761	
AST (≥40/<40 U/l)	48 (3–494)	0.004	0.316	
Alkaline phosphatase (≥80/<80 U/l)	84 (26–405)	<0.001	<0.001	1.552 (1.213–1.984)
GGT (≥80/<80 U/l)	103 (7–879)	0.002	0.113	
ALBI (≤-2.60/≥-2.60,<-1.39/≥-1.39)	231/167/3 (57.6/41.6/0.8%)	0.847		
RBC (≥5.7/<5.7×10^12^/L)	4.7 (1.3–7.8)	0.143		
WBC (≥6.5/<6.5×10^9^/L)	6.7 (2.5–17.3)	0.114		
PLT (≥174/<174×10^9^/L)	213 (48–720)	0.197		
AFP (≥400/<400ng/ml)	241/160 (60.1/39.9%)	0.245		
Child-Pugh (A/B)	393/8 (98/2%)	0.744		
Cirrhosis (yes/no)	209/192 (52.1/47.9%)	0.861		
Tumor number(>1/1)	169/232 (42.1/57.9%)	<0.001	0.004	1.406 (1.116–1.772)
Tumor size (≥10/<10cm)	10 (1.5–25)	0.044	0.864	
Tumor capsule (complete/ incomplete)	105/296 (26.2/73.8%)	0.001	0.003	1.531 (1.157–2.024)

Abbreviations: AFP, alpha-fetoprotein; ALBI, albumin-bilirubin; ALT, alanine transaminase; AST, aspartate aminotransferase; BDTT, bile duct tumor thrombus; GGT, gamma-glutamyl transpeptidase; HVTT, hepatic venous tumor thrombus; PLT, platelet; PT, prothrombin time; PVTT, portal vein tumor thrombus; RBC, red blood cell; TBIL, total bilirubin; WBC, white blood cell.

**Table 2:** Basic characteristics of the validation cohort

Variable	No. of patients	Hazard ratio (95%CI)	*P*
Age (≥50 vs. <50 yrs)	47 (24–76)	1.223 (0.706–2.119)	0.474
body mass index (≥18.5 vs.<18.5)	22.5 (17.3–34.2)	2.349 (1.061–5.203)	0.035
Alkaline phosphatase (≥80 vs. <80 U/l)	79 (23–729)	3.405 (1.910–6.071)	<0.001
Tumor number (>1 vs. 1)	39/123	2.559 (1.443–4.537)	0.001
Tumor capsule (complete vs. incomplete/absent)	37/125	2.159 (1.018–4.580)	0.045
Prognostic points (≤3.5 vs. >3.5)	113/49	1.163 (1.379–1.886)	0.003

### Univariate analysis, multivariate analysis and subgroup analyses

Univariate analysis showed that age, BMI, ALT, AST, ALP, GGT, tumor number, tumor size and tumor capsule correlated with overall survival (Table 1). In multivariate analysis, age (<50), BMI (<18.5), ALP (≥ 80), number of tumors (>1) and tumor capsule (incomplete) were independent risk factors for overall survival (Table 1). Subgroup analyses showed that age (<50 years), BMI (<18.5 kg/m^2^), ALP (≥80 U/l), tumor number (>1) and tumor capsule (incomplete) were associated with lower overall survival rate (Table 3). The incidence of portal vein tumor thrombus in patients less than 50 years old was 84%, which was significantly higher than that in patients older than 50 years old (62.5%; *P* <0.001).

**Table 3:** Median survival times and prognostic points of 401 patients with advanced hepatocellular carcinoma

Variable	No. of patients	Median survival time (months)	*P*	β	Points
Age (≥50 vs. <50 years)	165/236	18/10	0.013	0.242	1
body mass index (≥18.5 vs. <18.5)	358/43	13/8	0.031	0.467	2
Alkaline phosphatase (≥80 vs. <80 U/l)	221/180	11/19	<0.001	0.439	2
Tumor number (>1 vs. 1)	169/232	11/15	<0.001	0.341	**1.5**
Tumor capsule (complete vs. incomplete/absent)	105/296	20/11	0.001	0.426	2
Prognostic points (≤3.5 vs. >3.5)	177/224	23/9	<0.001		

### Prognostic scoring system

**Figure 1:**
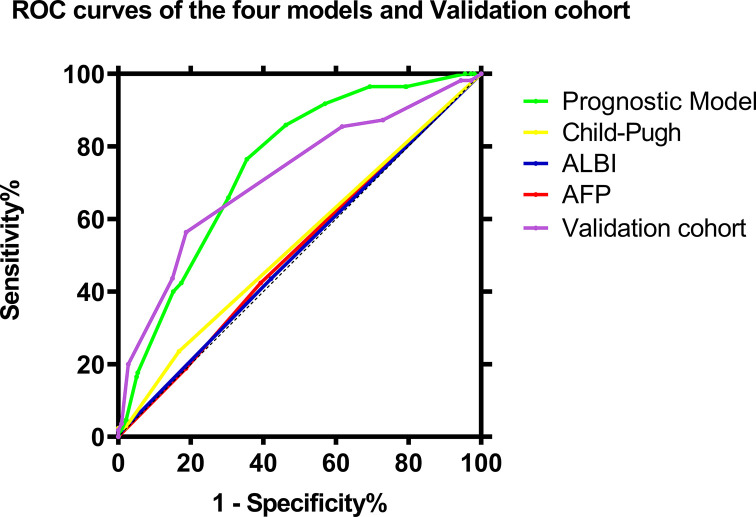
Survival curves of high-risk and low-risk groups ROC curves of the five models to predict patients’ overall survival after resection: the prognostic scoring system, alpha-fetoprotein (AFP), Child-Pugh, albumin-bilirubin (ALBI), and the validation cohort.

**Figure 2:**
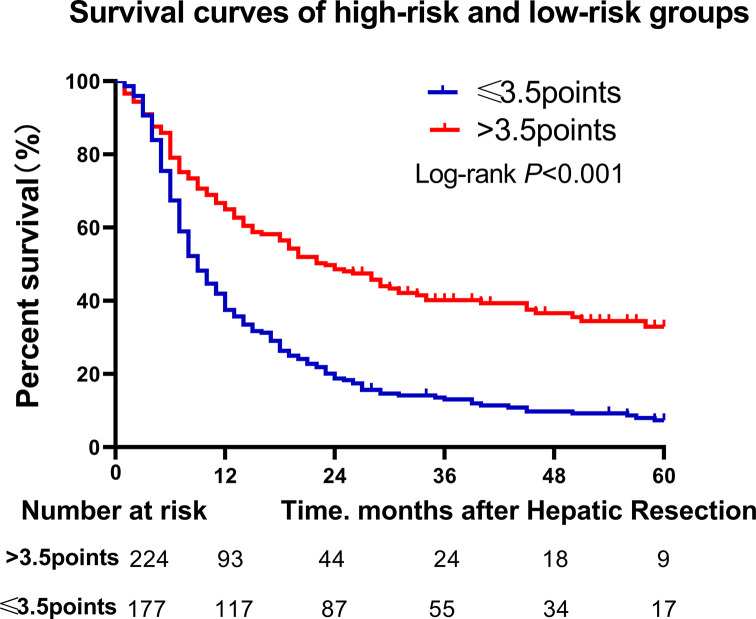
Survival curves of high- (score>3.5) and low-risk (score≤3.5) groups

Cox regression coefficients (β) were multiply by a factor of 4 and round to the nearest unit to facilitate our prognostic point calculation. The final model was prognostic point = age (≥50 years = 0, < 50 years = 1) + BMI (≥18.5 kg/m^2^ = 0, <18.5 kg/m^2^ = 2) + ALP (<80 U/l = 0, ≥80 U/l = 2) + tumor number (1 = 0, >1 = 1.5) + tumor capsule (complete = 0, incomplete = 2). According to the Youden index, a point of 3.5 was selected as the cut-off for categorizing patients as being at low-risk (≤3.5) or high-risk (>3.5) of poor overall survival after surgery, this cut-off gave sensitivity of 64.6% and specificity of 76.5%. The area under the receiver operating characteristic curve (AUC) of the scoring system was 0.747 (95%CI: 0.694–0.801, Figure 1), which is significantly better than some existing scoring systems, such as AFP (AUC = 0.520, 95% CI: 0.452–0.588, Figure 1), Child-Pugh (AUC = 0.547, 95% CI: 0.477–0.617, Figure 1) and ALBI (AUC = 0.536, 95% CI: 0.467–0.604, Figure 1). Low-risk patients showed higher overall survival rates at 1 year (65.0 vs. 37.5%), 3 years (40.2 vs. 13.0%) and 5 years (32.9 vs. 7.3%) (Figure 2). The AUC of validation cohorts was 0.716 (0.63–0.803, Figure 1), which has a good prediction accuracy.

### Overall survival

The median survival of training cohort was 12 ± 1.07 months, and validation cohort was 15 ± 1.63 months. In training cohort, by the end of follow-up, 316 patients had died and 85 survived; the rate of overall survival was 49.6% at 1 year; 25.0% at 3 years and 18.0% at 5 years; and the median survival in high-risk patients was 9 months, compared with 23 months in low-risk patients (Figure 2).

## Discussion

Official guidelines recommend systemic treatment with sorafenib or lenvatinib for advanced patients [4,22], yet many such patients are successfully treated by resection at medical centers around the world [7]. Prognostic models have been developed to predict recurrence or mortality after resection or TACE [23,24] but not necessarily to identify the patients more likely to benefit from resection or TACE. We found that younger age (<50 years), lower BMI (<18.5 kg/m^2^), higher ALP (≥ 80 U/l), larger number of tumors (>1) and incomplete tumor capsule were independent risk factors of poor overall survival after tumor resection. The present study establish a prognostic scoring model to select suitable advanced HCC patients who would benefit from resection.

BMI less than 18.5 kg/m^2^ is defined as underweight by the World Health Organization [25], and it is seen in many patients with advanced HCC. Our finding that lower BMI is associated with shorter survival in advanced HCC patients is consistent with studies showing that underweight is associated with larger tumors, poorer differentiation, macrovascular invasion, high AFP (>400 ng/ml), poor liver function, muscular dystrophy and tumor recurrence, all of which are predictors of poor prognosis in HCC [26–28].

We found that advanced HCC patients younger than 50 years may have shorter survival after resection. This result is consistent with a previous study showing that patients younger than 50 years had more HBV infection, more tumors, larger tumors, and later stage [29–32]. In our study, the incidence of portal vein tumor thrombus was higher in young patients, which may lead to poor prognosis.

Local blockage of intra- or extra-hepatic bile ducts often leads to increased ALP. High ALP (>200 ng/ml) is considered to increase risk of poor prognosis in the Chinese University Prognostic Index [33]. In our study, the cut-off value of ALP was 80 U/l, which may allow more sensitive detection of at-risk patients, possibly because all patients in the present study had advanced HCC. Our result is consistent with a previous study showing that ALP > 82 U/l was associated with poor prognosis and was a better predictor of poor prognosis than AFP levels [34]. Shorter survival in advanced HCC patients with high ALP may be due to the ability of ALP to promote proliferation, invasion and metastasis [35].

It is well known that incomplete tumor capsule and larger tumor number adversely impact overall survival in HCC patients [36,37]. Our results support this idea. Short survival in advanced HCC patients with incomplete tumor capsule and multiple tumors may be related to increased risk of microvascular invasion and metastasis [38,39].

In the present study, poor prognosis was not found to be related to patients with large vascular tumor thrombus. This may be because all patients in the present study had advanced HCC, while few had type III tumor thrombus [40]. This may also be because tumor thrombi were surgically removed in all patients. For patients with advanced hepatocellular carcinoma, this model has better recognition ability than ALBI, Child-Pugh and AFP. In addition, this model includes the patient’s physical status, biochemical indicators, and tumor characteristics, fully considering the influence of preoperative indicators on prognosis. The inclusion criteria of our model are patients with advanced liver cancer. Variables in the model are common, which can be verified by many published models and have wide availability.

When patients with advanced hepatocellular carcinoma are considering surgery, our research can provide reference for treatment. The median survival of low - and high-risk patients in our study can be compared to patients on other treatments.

According to our research, we recommend surgical treatment for low-risk patients, the median survival time of patients undergoing surgery is 23 months, and the prognosis is better than that of patients who only receive systematic treatment [41].

Surgical treatment for high-risk patients needs to be carefully considered, and the prognosis of patients undergoing surgical treatment is worse than that of patients with systematic treatment [41]. At present, after conversion therapy is effective, surgical treatment has also achieved a good prognosis, but more researches are needed to determine whether this treatment regimen will benefit all high-risk patients [42].

There are a few limitations in the present study. First, this was a single-center retrospective study, so the results may not be generalizable to other patient populations. Second, the prognostic scoring system does not take into account postoperative interventions such as systemic therapy or adjuvant TACE or radiotherapy, which can prolong survival in advanced HCC patients [43–45]. Despite these limitations, the present study demonstrates the feasibility of predicting advanced HCC patients who are more likely to benefit from resection, even based solely on preoperative variables such as age, BMI, ALP, number of tumors and tumor capsule. The present study establishes a prognostic scoring model to select suitable advanced HCC patients who would benefit from resection.

## Data Availability

The clinical data used to support the findings of this study are included within the article.

## Abbreviations

AFP: alpha-fetoprotein
ALBI: albumin-bilirubin
ALT: alanine transaminase
AST: aspartate aminotransferase
BDTT: bile duct tumor thrombus
GGT: gamma-glutamyl transpeptidase
HCC: Hepatocellular carcinoma
HVTT: hepatic venous tumor thrombus
PLT: platelet
PT: prothrombin time
PVTT: portal vein tumor thrombus
RBC: red blood cell
TBIL: total bilirubin
WBC: white blood cell
